# Pharmacophore- and QSAR-guided discovery of natural product inhibitors targeting dehydrosqualene synthase in *Staphylococcus aureus*

**DOI:** 10.1371/journal.pone.0345620

**Published:** 2026-04-29

**Authors:** Juliana Amorim, Santiago Ortega, Ricardo Rodas, Luis Vélez, Denisse Arteaga, Juan Carpio

**Affiliations:** Academic Unit of Health and Wellness, Universidad Católica de Cuenca, Azuay, Ecuador; University of Nairobi Faculty of Health Sciences, KENYA

## Abstract

Dehydrosqualene synthase is a validated antibacterial target involved in *Staphylococcus aureus* staphyloxanthin biosynthesis, a key factor in oxidative stress resistance and virulence. From a dataset of 299,845 natural products, we applied pharmacophore-based screening followed by QSAR-based screening, identifying 1,978 compounds with predicted *Ki* values of 100 nM or lower. Among them, 47 molecules showed remarkable predicted activity, with *Ki* values lower than 1.0 nanomolar. Notably, despite the wide chemical diversity of the library evaluated and the fact that the models were not trained with certain scaffolds of known inhibitors of staphyloxanthin biosynthesis, nine of these chemotypes were recovered among these top candidates, demonstrating the ability of the models to capture relevant structural features beyond the training data. Five representative compounds from this group showed high binding affinity and stable interactions with key CrtM residues in molecular dynamics simulations, reflecting the potential of the overall set as a source of promising leads for novel antivirulence agents targeting dehydrosqualene synthase in *S. aureus*.

## Introduction

Therapeutic strategies aimed at inhibiting bacterial virulence factors have become a major focus in antimicrobial research [1]. *Staphylococcus aureus* is among the bacterial species that produce numerous such factors [2]. This clinically significant pathogen is capable of causing a broad spectrum of human infections and can persist in diverse host environments, ranging from skin surfaces to abiotic medical devices and deep-seated tissues [3,4]. Its adaptability and persistence make it challenging to eradicate, highlighting its potential as a target for the development of novel therapeutic approaches [5].

One of the main virulence factors of *S. aureus* is staphyloxanthin (SXT), a carotenoid pigment that plays an essential role in its defense against oxidative stress [4]. Furthermore, although not part of classical quorum sensing, STX has been reported to confer cross-species protection by transferring from *S. aureus* to *Pseudomonas aeruginosa*, thereby enhancing the resistance of the latter to oxidative damage [6]. In murine models of type 2 diabetes, pigment-producing *S. aureus* strains exhibited enhanced immune evasion and significantly delayed wound healing, whereas STX-deficient mutants were more susceptible to host defenses and allowed faster tissue recovery [7].

The biosynthesis of SXT begins with the precursor molecule farnesyl diphosphate (FPP), which undergoes a series of enzymatic transformations [8]. A key enzyme in this pathway is dehydrosqualene synthase (CrtM), which catalyzes the head-to-head condensation of two FPP molecules to produce dehydrosqualene. This reaction initiates the formation of the triterpenoid backbone that serves as the structural precursor for STX. Following dehydrosqualene synthesis, the biosynthetic pathway proceeds through a cascade of reductive and oxidative enzymatic modifications, ultimately leading to the production of STX [9].

Numerous experimental studies have demonstrated that impaired STX production results in increased bacterial susceptibility to oxidative stress and a marked attenuation of pathogenic traits. Compounds that suppress STX production include several natural products including both microbial-derived metabolites [10] and phytochemicals [8,9]. Celastrol, a plant triterpenoid, reduces STX production by 50% at 0.36 µM (approximately 0.16 µg/mL) [11]. Flavone, at a concentration of 50 µg/mL, suppressed STX synthesis and increased the susceptibility of *S. aureus* to hydrogen peroxide by approximately 100-fold [12]. In contrast, thymol required a concentration of 100 µg/mL to achieve a 90% reduction in STX production [13].

CrtM has been experimentally validated as a molecular target through multiple inhibitors with known *Ki* values [14]. These quantitative data make CrtM particularly suitable for ligand-based drug discovery, in contrast to other targets in this pathway for which fewer validated ligands are available [15]. However, it is important to note that certain reported compounds reducing STX levels may operate through mechanisms beyond direct enzyme inhibition. In some cases, pigment suppression may arise from transcriptional regulation or from the inhibition of other enzymes within the biosynthetic pathway, rather than from direct interference with CrtM activity [5,8,16]. For example, the natural monoterpenoid myrtenol reduced STX levels by approximately 20% at 75 μg/mL by downregulating CrtM expression [17]. Similarly, in another study, carvacrol at the same concentration (75 µg/mL) inhibited 72% of STX production and its metabolic intermediates in methicillin-resistant *S. aureus* (MRSA) through downregulation of CrtM gene expression [18].

As the chemical space of confirmed CrtM inhibitors remains relatively limited, the structural diversity of natural products provides a valuable opportunity to discover candidates with improved potency and pharmacological properties, potentially more suitable for clinical development than those reported to date. A major limitation, however, lies in the uncertainty surrounding the molecular targets of many reported compounds, as numerous studies rely solely on SXT reduction as a phenotypic readout without experimentally confirming direct inhibition of the enzyme [8,10]. This challenge hinders the accurate selection of candidates with genuine CrtM inhibitory activity. In this context, *in silico* drug discovery provides structure- or mechanism-based strategies for the early identification and rational optimization of compounds [19,20]. In fact, a recent *in silico* study employed machine learning–based QSAR models to evaluate 1,261 natural compounds as potential CrtM inhibitors, ultimately finding several promising candidates despite the relatively limited number of molecules assessed [21].

In this context, the present study aimed to identify natural compounds with potential CrtM inhibitory activity by implementing an integrated *in silico* strategy. Using two sequential virtual screening strategies—pharmacophore- and QSAR-based—were integrated with molecular dynamics analyses to identify promising candidates from a library of 299,845 natural products. This approach led to the identification of structurally diverse molecules with the potential to inhibit CrtM at concentrations predicted to be comparable to those of the most potent inhibitors reported to date, and exhibiting overall acceptable pharmacokinetic and toxicity profiles for hit compounds. Overall, the results presented here provide a valuable basis for the development of novel anti-virulence agents targeting *S. aureus*.

## Methodology

### Selection of active compounds and generation of decoys

In the BindingDB database [22], 93 compounds were reported as CrtM inhibitors; among them, only 35 compounds had experimentally determined *Ki* values, ranging from 0.2 to 20,160 nM (corresponding to a p*Kᵢ* range of approximately 9.70 to 4.70), and these were used for the QSAR model construction. A subset of the 22 most potent inhibitors was selected for pharmacophore model generation. Decoy compounds were generated using the DUD-E web server [23], with the SMILES of each active molecule provided as input.

### In silico compounds preparation

A total of 299,845 natural product structures were retrieved from the COCONUT database (COlleCtion of Open Natural prodUcTs) [24] and prepared for computational analysis using RDKit, Pandas, and NumPy libraries to generate 3D structures and perform file editing. Protonation states were standardized to pH 7.4, and geometry optimization was conducted through energy minimization with the MMFF94 force field. The process employed the steepest descent algorithm followed by the conjugate gradient method with default parameters, as implemented in Open Babel v3.1.1 [25]. All the structures were then converted to MOL2 and SDF formats.

### Pharmacophore modeling, validation, and screening

A ligand-based pharmacophore model was developed using a dataset composed of diverse CrtM inhibitors and their corresponding decoys. The model was constructed using the PSearch software [26], which employs a three-dimensional pharmacophore signature approach independent of molecular alignment. Unlike other methods, PSearch does not divide the dataset into training and test subsets; instead, pharmacophore hypotheses are derived from the active molecules and statistically evaluated using the F-score (F₀.₅). Within PSearch, decoy molecules are incorporated to assess the discriminative performance of each model between active and inactive compounds, while compound clustering reduces redundancy and mitigates potential overfitting. This approach ensures that the resulting pharmacophores capture activity-related structural features rather than general physicochemical properties [26]. In the modeling protocol, referred to as “Strategy 1” within the software, the 22 most potent active compounds—with experimentally determined activities up to 1000 nM—were selected from the previously described set of 35 molecules and compiled into a training set used for model generation. The remaining molecules were excluded to avoid introducing noise and biasing the model toward less promising chemical profiles. To enhance the chemical diversity of the active set, the co-crystallized reference ligand (compound 673) was included. This compound is reported in the Protein Data Bank under the entry PDB ID: 3ACX, which corresponds to the experimentally resolved crystal structure of the CrtM–ligand complex.

Validation of the pharmacophore model was carried out using a dataset of 270 compounds, consisting of active molecules and 248 decoys. Each compound was screened using the final pharmacophore hypothesis in the Pharmit web server [27]. Pharmit only returns compounds that satisfy all pharmacophoric constraints; these were assigned a binary score of 1 (match), whereas all non-retrieved compounds were assigned a value of 0 (non-match). These binary outputs were compared with the true activity labels (1 for actives and 0 for decoys) to evaluate the ability of the pharmacophore model to distinguish between the two classes.

Sensitivity was defined as the proportion of actives correctly identified by the model (true positives, TP, divided by TP + false negatives, FN), whereas specificity represented the proportion of decoys correctly rejected (true negatives, TN, divided by TN + false positives, FP). Overall accuracy was computed as (TP + TN)/*N*, where *N* is the total number of compounds in the validation set. The ROC curve and its corresponding area under the curve (AUC) were derived directly from the binary match scores.

Early enrichment was assessed by determining how many active compounds appeared in the top-ranked subset relative to the number expected by chance, following the standard definition of enrichment factor used in decoy-based virtual screening workflows [28]. The expected value was calculated using the expression (*k/N*) × *A*, where *k* is the number of compounds in the top-ranking fraction, *N* is the total number of screened molecules, and *A* is the total number of actives. The enrichment factor (EF) was obtained by dividing the number of actives retrieved in the top subset by this expected value.

Structural similarity analysis was carried out using SkelSpheres fingerprints to evaluate the chemical diversity within the set of 23 active compounds employing DataWarrior software [29]. Once the pharmacophore model meeting the statistical criterion (F₀.₅ ≥ 0.8) was obtained, it was used for virtual screening on the Pharmit server against the library of 299,845 natural products. A molecular weight filter of 300–500 Da was applied to select compounds within the drug-like range for further analysis.

### Quantitative structure-activity relationship modeling

QSAR models were developed and validated using the QSARINS software package (version 2.2.4) [30], which applies a genetic algorithm (GA) to iteratively select the descriptors most closely associated with the observed activity. Final model construction was performed using multiple linear regression (MLR), and performance was thoroughly evaluated using statistical parameters to guarantee both robustness and predictive accuracy.

The data set of 35 active molecules were divided into a training set of 25 and a test set of eight, using the response-based split option in QSARINS to preserve a broader range of activity values. Two of the active molecules were excluded for being outside the applicability domain during QSAR model construction. The experimental *Ki* values of the actives, expressed in nanomolar concentrations, were converted to p*Ki* values. Molecular descriptors were computed using the OCHEM online platform (version 4.3.156) [31], with compound structures provided in SDF format. The pool of descriptors considered for model development were generated with alvaDesc v.2.0.16 (5,666 descriptors), Dragon v.7 (5,270 descriptors), CDK 2.8 (256 descriptors), RDKit and MORDRED (1,826 descriptors). Additional descriptor sets were obtained from MOPAC2016 (35 descriptors), KrakenX (124 MOPAC 2016-derived descriptors), *Py*Descriptor (16,251 descriptors), MERA (529 descriptors), MERSY (42 descriptors), Inductive descriptors (54 descriptors), and Spectrophores (144 descriptors). Molecular structures were geometry-optimized using ULYSSES [32], a C++ library for semiempirical quantum chemical calculations, integrated within the OCHEM platform. The PM6 semiempirical method was used to generate low-energy conformations [33]. To refine the descriptor pool, features that were constant in more than 80% of the molecules or exhibited pairwise correlations above 95% were removed, yielding a final set of 1,783 descriptors. Variable selection was carried out using a genetic algorithm (GA) with a population size of 20, a maximum of 10,000 generations, and a mutation rate of 20%.

### QSAR model validation

The reliability of QSAR models is determined not only by their ability to reproduce the experimental data but also on demonstrating robust predictive capacity through comprehensive validation protocols [34]. To verify the internal validity and robustness of the models, several cross-validation strategies were employed, including leave-one-out (Q²_LOO), leave-many-out (Q²_LMO), and the Concordance Correlation Coefficient in cross-validation (CCC_cv). The LMO procedure was repeated over 2,000 iterations, each time randomly excluding 30% of the dataset. In addition, Y-randomization tests were carried out to assess the likelihood of chance correlation; this involved scrambling 30% of the response values over 2,000 iterations and evaluating model performance under these randomized conditions. To test external predictive power, independent test set predictions were analyzed using Q²_ext-F1, Q²_ext-F2, Q²_ext-F3, and the external Concordance Correlation Coefficient (CCC_ext). The applicability domain (AD) was defined via the leverage method, and visualized using a Williams plot, allowing the detection of compounds that fall outside the reliable prediction space of the model. The warning leverage threshold was established based on the leverage formula applied to the training set h* = 3(*p* + 1)/*n*, where h* is the critical leverage value, *p* is the number of descriptors, and *n* is the number of molecules in the training set [35].

For the QSAR-driven virtual screening, we calculated the molecular descriptors included in the final model equation for each compound and used them to generate pKi predictions. To assess whether the selected compounds were within the applicability domain (AD) defined by the training set, the Insubria graph was used as a visual analyses tool [36].

### Similarity analyses

Structural similarity of the molecules in the datasets was evaluated using DataWarrior software (version 060402) [29], employing the FragFp fingerprint algorithm. This approach enabled the identification of fragment-level similarities by encoding characteristic functional groups and substructural patterns. The algorithm is based on a curated set of 512 frequently occurring fragments in organic compounds, designed to reduce redundancy while preserving chemical relevance. The inclusion of wildcard atoms in many fragments enables FragFp to detect meaningful structural similarities even in the presence of small atomic differences, making it particularly effective for comparing diverse scaffolds such as those in our dataset [29]. In addition, similarity analysis based on SkelSpheres fingerprints was also performed to assess topological relationships among compounds. Unlike FragFp, SkelSpheres focuses on the two-dimensional connectivity of the molecular framework, providing a complementary measure of structural diversity within the dataset [29].

### In silico toxicity and drug-likeness evaluation of the best-scoring CrtM candidates

The toxicity profile of the best-scoring CrtM inhibitors was evaluated using the pkCSM web server [37], which predicts pharmacokinetic and toxicity parameters based on graph-based molecular signatures derived from the compound structure. SMILES strings of the selected ligands were submitted to the pkCSM interface to estimate endpoints relevant to early drug safety assessment, including mutagenicity (Ames test), hepatotoxicity, skin sensitization, hERG I and II inhibition, and systemic toxicity parameters such as the maximum tolerated dose (MTD), oral rat acute toxicity (LD₅₀), and chronic oral toxicity (LOAEL). The resulting predictions were analyzed comparatively among the selected compounds to identify relative differences in toxicity risk.

The druglikeness, medicinal chemistry, and pharmacokinetic profiles of the top-ranked CrtM inhibitor candidates were evaluated using the SwissADME web tool [38]. Each compound was submitted in SMILES format to calculate physicochemical and ADME-related parameters. The assessment included druglikeness filters such as Lipinski, Ghose, Veber, Egan, and Muegge rules, along with the bioavailability score. Medicinal chemistry parameters included PAINS and Brenk structural alerts, leadlikeness, and synthetic accessibility. The consensus Log Po/w was used to estimate lipophilicity, while gastrointestinal (GI) absorption and blood–brain barrier (BBB) permeability predictions provided insights into pharmacokinetic behavior.

### Molecular docking analysis

Following the QSAR-based virtual screening, molecular docking was carried out to determine the binding modes of the most promising ligands within the CrtM active site. The resulting complexes provided the initial coordinates for the subsequent molecular dynamic simulations. Docking analyses were performed using the Protein-Ligand ANT System v1.2 (PLANTS) [39]. For this purpose, the crystal structure of CrtM with the identifier PDBe ID: 3ACX [40] retrieved from the Protein Data Bank in Europe – Knowledge Base (PDBe-KB) [41], was selected as the target structure. Protonation states were assigned at pH 7.4 using PropKa v3.0 [42], with all other parameters set to default. The prepared structure was then processed with the SPORES v1.3 script under default settings and saved in MOL2 format. All docking runs were carried out within a 12.5 Å radius centered on the co-crystallized ligand, using coordinates (x = 55, y = 15, z = 55). An RMSD threshold of 2.0 Å was applied to ensure effective clustering, while other parameters remained at their default values.

### Molecular dynamic simulations

All molecular dynamics (MD) simulations of the enzyme–ligand complexes were carried out using GROMACS v2024.4 [43], with the all-atom CHARMM36m force field [44]. The TIP3P water model was employed for all systems, placed within a periodic triclinic box maintaining a minimum distance of 1.0 nm from the solute to the box edge. To mimic physiological ionic strength, Na⁺ and Cl⁻ ions were added to achieve a 0.15 mol/L concentration. Energy minimization was performed using the steepest descent algorithm until the maximum force converged below 500 kJ/mol, eliminating steric clashes. System equilibration was conducted in two phases: first, a 1 ns NVT simulation gradually increasing the temperature to 310 K using the V-rescale thermostat; second, a 3 ns NPT simulation at 1 bar with the C-rescale barostat. Production MD runs were then performed for 500 ns under the Parrinello–Rahman barostat. Long-range electrostatic interactions were computed using the Particle Mesh Ewald (PME) method [45] and the hydrogen bond constraints were applied using the LINCS algorithm [46].

### Binding free energy calculations and interaction analyses

Calculations were performed using the MD trajectories to assess the total binding free energy, and to further analyze the interactions influencing the total energy of the selected complexes. For total binding free energy calculations, the MMPBSA methodology [47] was employed, utilizing a single trajectory and performed with the gmx-MMPBSA software (version 1.6.0) [48]. From the MD results of all runs, 500 snapshots were extracted for total energy and amino acid energy decomposition analysis. All settings followed the recommended default values of the software.

**Fig 1:**
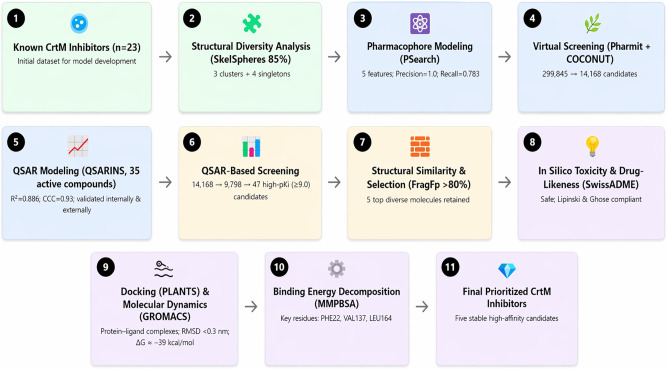
Workflow of the computational strategy for identifying novel CrtM inhibitors. Sequential methodological pipeline applied for the discovery and prioritization of potential CrtM inhibitors from natural products. The workflow integrates ligand-based pharmacophore modeling, QSAR model generation, validation and virtual screening, structural similarity analysis, and *in silico* ADMET evaluation, followed by molecular docking and molecular dynamics simulations. Each numbered step (1–11) corresponds to a specific stage of the computational protocol, showing the progression from known active ligands to the final selection of five stable and high-affinity CrtM inhibitor candidates.

The overall computational workflow employed in this study is summarized in Fig 1.

## Results and discussion

### Pharmacophore modeling

**Fig 2:**
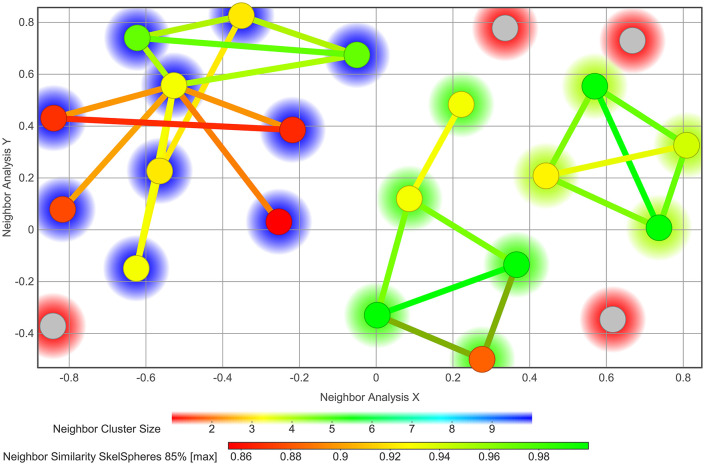
Structural diversity of the CrtM inhibitors used for pharmacophore modeling. SkelSpheres fingerprint analysis at an 85% similarity threshold grouped the 23 active inhibitors into three major clusters containing ten, five, and four molecules, respectively, and four singletons (grey nodes) with similarity values below the cutoff.

**Fig 3:**
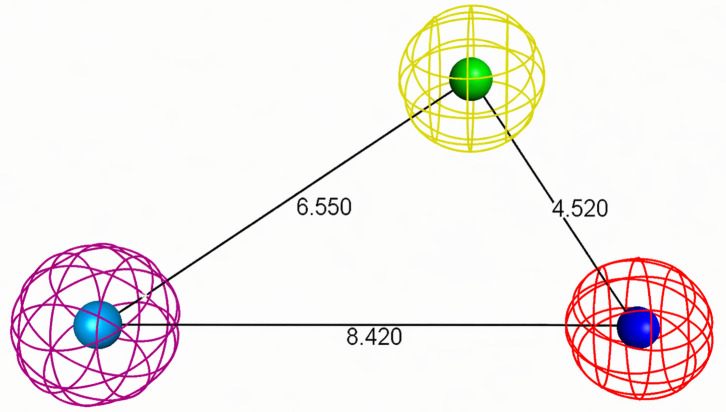
Pharmacophore model 3D representation. Magenta sphere represents an aromatic feature spatially overlapping with a hydrophobic feature. Yellow and red spheres correspond to two hydrogen bond acceptor features, with red sphere overlapping with the negative ionizable feature. Distances between features (Å) are given by numerical labels, measured between feature centroids.

A ligand-based pharmacophore model was constructed to identify and prioritize the most promising candidates from the extensive natural product database [49]. To construct the pharmacophore model, 23 potent CrtM inhibitors from the dataset of 35 active molecules were selected to capture the essential chemical features underlying their inhibitory activity (S1 and S2 Tables). A structural diversity analysis using SkelSpheres fingerprints with an 85% similarity threshold revealed that these compounds were distributed into three main clusters containing ten, five, and four molecules, respectively, along with four singletons showing similarity values below the defined cutoff (Fig 2). Furthermore, within the largest cluster, several molecules display marked structural differences, as shown by the orange and red nodes and edges. This clustering pattern indicates that the active set shares coherent chemotypes with reproducible structural features, while still retaining enough peripheral diversity to prevent overfitting to a single scaffold. The pharmacophore model generated with PSearch from these inhibitors exhibited robust predictive performance across all statistical metrics, including a precision of 1.0 and a recall of 0.783 (S3 Table). This pharmacophore harbors five features, each with a 1.0 Å radius: an aromatic feature at coordinates (1.21, 0.14, 0.48) Å; two hydrogen bond acceptors at (−6.76, 1.75, −1.49) Å and (−4.08, −1.47, −3.02) Å; a negative ionizable feature at (−6.76, 1.75, −1.49) Å; and a hydrophobic feature at (1.21, 0.14, 0.48) Å (Fig 3).

The validation screening was performed in Pharmit. Although 23 active molecules were initially considered, one of them could not be included because it was not compatible with the decoy-generation procedure, resulting in a final validation set of 22 actives. Of these, the pharmacophore model successfully retrieved 20, while only 33 of the 248 decoys matched the pharmacophoric features. This corresponded to a sensitivity of 0.909 and a specificity of 0.867, with an overall accuracy of 0.870. The ROC analysis yielded an AUC of 0.888, reflecting strong discriminative power even with binary match scores. Early enrichment analysis further supported the effectiveness of the model: 20 actives appeared within the top 53 ranked compounds (~19.6% of the dataset), whereas only 4.3 would be expected by chance alone, resulting in an enrichment factor of 4.64. The statistical performance of the pharmacophore model is consistent with the ranges typically reported for ligand-based pharmacophore models employing actives–plus–decoys validation [50,51]. The AUC value of 0.888 is particularly noteworthy, as AUC values above 0.85 are generally interpreted as good evidence of discriminative power in decoy-based validation frameworks, as demonstrated in pharmacophore-guided virtual screening studies employing similar evaluation criteria [28]. Similarly, the enrichment factor (EF = 4.64) reflects meaningful early recognition performance, which is a key aspect of decoy-based validation in pharmacophore-guided virtual screening. Although the number of available active compounds was moderate the strong external validation performance, indicates that the model captures meaningful activity-related features and mitigates potential bias arising from dataset size.

Using this pharmacophore as input in the Pharmit web server, 299,845 natural products from the COCONUT database were screened, resulting in 14,168 candidate inhibitors selected for subsequent analyses (S4 Table).

### QSAR modeling

To predict the *Ki* values of the previously identified molecules, a QSAR model was developed in QSARINS using the complete dataset of 35 inhibitors with experimentally determined *Ki* values. While other CrtM inhibitors have been reported in the literature, most are described through IC₅₀ or percent inhibition data rather than *Ki*. The latter was preferred because it represents a direct and assay-independent measure of binding affinity, ensuring a more consistent foundation for QSAR modeling [52]. The final dataset included 25 molecules for training and 8 for external validation, whereas two compounds were outside the applicability domain. The final model was constructed using three molecular descriptors to maintain a balanced descriptor-to-sample ratio and reduce the risk of overfitting [35]. These molecular descriptors were: AETA_eta_B, NDLIB22 and N_O_3A. The model equation is: Ŷ = 6.7117 + 95.8573 × AETA_eta_B – 0.199 × NDLIB22–0.9159 × N_O_3A. The AETA_eta_B descriptor (calculated by Mordred) is the averaged ETA branching index, which captures molecular branching within the ETA (Extended Topochemical Atom) framework, reflecting how atomic connectivity and topochemical contributions distribute across the structure [53,54]. Its positive coefficient suggests that, within the modeled chemical space, increased branching-related topochemical complexity is associated with enhance biological activity. NDLIB22 is a MERA descriptor [55] that quantifies the normalized spatial variance of atomic positions along a principal component of the geometry of the molecule. It reflects the extent of molecular elongation or dispersion along this axis, independent of overall size. Its negative coefficient suggests that greater spatial spread in this direction may reduce biological activity. N_O_3A is a topological atom-pair descriptor that counts the number of oxygen atoms located exactly three bonds from a nitrogen atom. It encodes topological proximity without inferring chemical functionality. Its negative coefficient suggests that such N–O arrangements are unfavorable for activity. This feature is easily observed in the less active molecules used to build the model.

Although all three descriptors included in the model are statistically significant (*p* < 0.001) (S5 Table), their relative contributions differ, as reflected in their standardized coefficients. The descriptors N_O_3A (–0.6237) and AETA_eta_B (+0.589) exhibited the highest absolute values, indicating that topological N–O relationships and ETA (topochemical) show the strongest influence on the predicted activity within this model. In contrast, NDLIB22 presents a smaller standardized coefficient (–0.2781), suggesting a weaker contribution to the predicted activity. However, this 3D descriptor complements the model by capturing geometric features not accounted for by the 2D descriptors.

**Fig 4:**
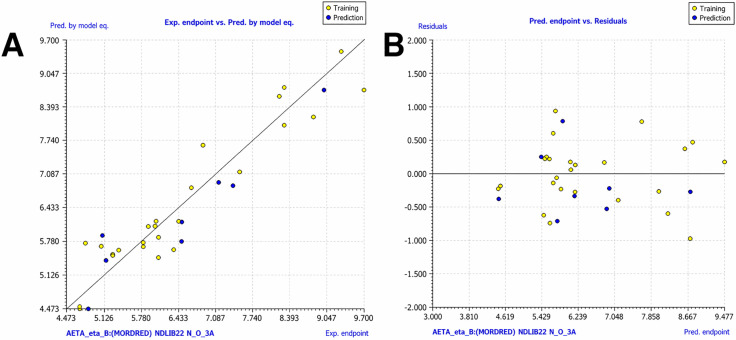
Experimental vs. predicted endpoint values for the QSAR model. **(A)** Scatter plot of experimental vs predicted p*Ki* by MLR and **(B)** Scatter plot of Predicted vs Residuals values.

The quality of the model was assessed using the coefficient of determination (R²). The obtained value of R² = 0.886 indicates that the model explains a substantial proportion of the variance in the experimental *Kᵢ* values of the compounds in the training set. The dispersion observed for a few compounds (Fig 4A) is within the expected range for QSAR models. Furthermore, the residuals vs. predicted plot shows residuals randomly scattered around zero, all within ±1, with similar scatter patterns between the training and prediction sets (Fig 4B), suggesting an absence of overfitting [35].

**Fig 5:**
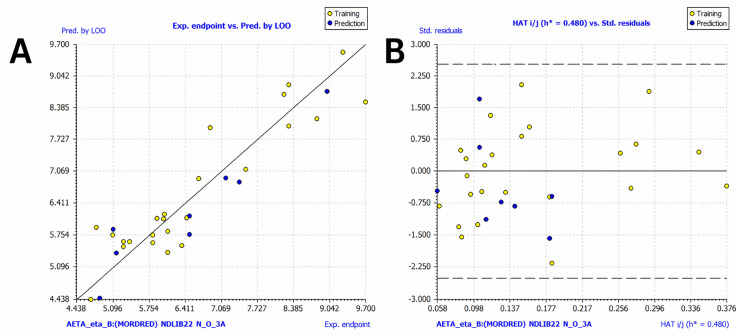
Internal validation and applicability domain assessment. **(A)** Scatter plot of experimental vs Q2-LOO (Leave-One-Out) validation. **(B)** Williams plot for selected model, with the vertical black line on the right indicating Hat ≤ h* = 0.480.

In the internal validation, assessed through Q^2^ using leave-one-out cross-validation (Fig 5A), each compound in the training set was systematically excluded and predicted by a model built without it. The predicted values from LOO exhibit close agreement with the experimental data, with most points distributed near the identity line. This strong correlation indicates both the robustness of the model and its ability to generalize within the chemical space of the training set. To evaluate the reliability of individual predictions and identify potential outliers, a Williams plot was constructed by plotting standardized residuals against leverage values (Fig 5B). All compounds from both the training and test sets were found to fall below the warning leverage threshold (h* = 0.480), confirming that the QSAR model operated entirely within its applicability domain and was not influenced by outliers or leverage values. Additionally, the standardized residuals remained within ±2.5 standard deviation units, indicating the absence of extreme outliers [56].

**Fig 6:**
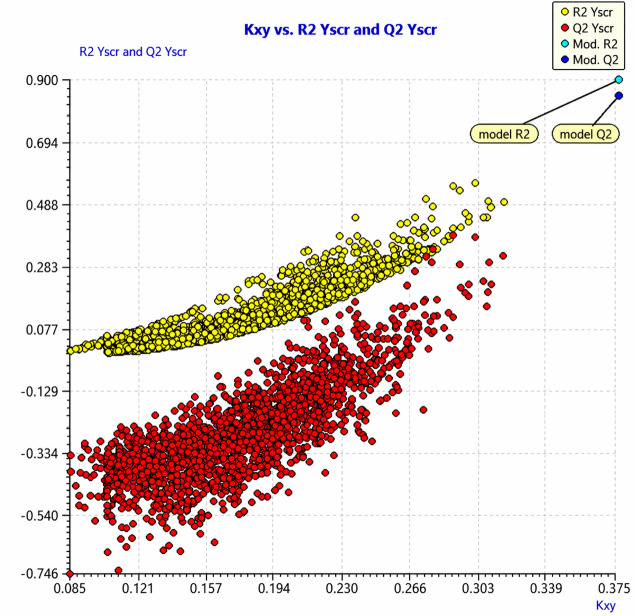
Y-scrambling analysis. 𝑅2 and 𝑅2 values from 2,000 iterations of Y-scrambled models (yellow and red dots) compared with the performance of the selected model (cyan and blue dots).

To further assess the robustness of the model and ensure that the observed correlations were not the result of chance, a Y-randomization test was performed using 2,000 iterations, with 30% of the response variable randomly scrambled in each iteration. The results show that R²_Yscr (yellow dots) and Q²_Yscr (red dots) values from the randomized models were markedly lower than the R² and Q² values of the original model (cyan and blue dots, respectively) (Fig 6), supporting the conclusion that the performance of the model is not due to chance correlations [36].

The predictive ability of the QSAR model was further assessed through external validation using an independent test set (S5 Table), in line with the requirement that predictivity be demonstrated on truly external compounds not used in model training [34]. The high coefficient of determination (R^2^ = 0.89) for the test set indicates that the model maintains a high level of predictive consistency when applied to external compounds. The predictive squared correlation coefficients across different formulations of the Q² metric: Q²-F1 (0.87), Q²-F2 (0.87), and Q²-F3 (0.89) all fall within acceptable thresholds, supporting the robustness of the model [57]. Additionally, the external concordance correlation coefficient (CCC = 0.93) demonstrates both precision and accuracy in the predictions [58,59]. Low values of RMSE (0.4802), MAE (0.43), and PRESS (1.84) reflect a good predictive performance, indicating limited error between observed and predicted activities, while the Δr² (0.11), calculated as the absolute difference between the training and test set R², suggests strong internal consistency and reduces concern about overfitting.

Altogether, the internal and external validation results, along with the Y-randomization test and applicability domain analysis, demonstrate that the developed QSAR model is robust, statistically reliable, and possesses strong predictive power within its defined chemical space.

### QSAR-based screening

In the next step, QSAR-based screening of the 14,168 compounds previously filtered by the pharmacophore yielded a subset of 9,798 structures. The remaining molecules were excluded as they fell outside the applicability domain and the activity range defined by the active compounds in the training set. Notably, 1,978 molecules exhibited values equal or below 100 nM (p*Ki* ≥ 7). Considering that, compounds with potencies in the 100 nM to 5 µM range are typically classified as hits in early screening stages [60], the predicted high affinities of these compounds provide a solid rationale for their prioritization in subsequent studies. Furthermore, 47 molecules were predicted to be highly active, with p*Ki* values ranging from 9.471 to 9.004 (corresponding to predicted *Ki* values of 0.338 to 0.989 nM, respectively, S6 Table).

### Analysis of structural similarity

**Fig 7:**
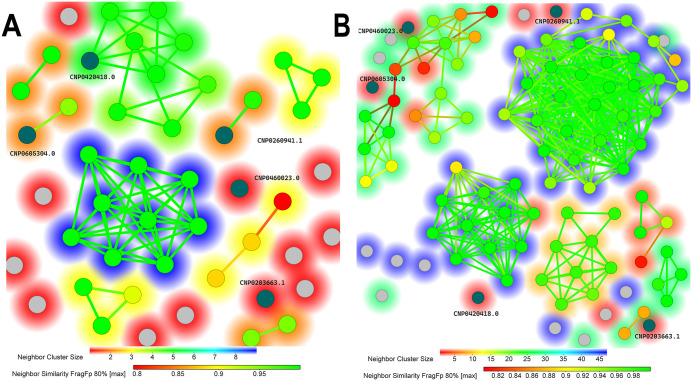
Similarity maps of the best-ranked compounds and reported inhibitors. **(A)** Network of the 47 best-ranked compounds, showing similarity relationships based on the *FragFp* fingerprint with an 80% similarity cutoff. **(B)** Network of inhibitors generated with the same grouping strategy. In both panels, each node represents a molecule, and edges connect pairs with similarity ≥ 0.80. Dark blue nodes represent the best-ranked compounds. Node colors reflect neighbor similarity values, ranging from red (0.80) to green (0.95), with higher similarity values forming tighter clusters. Grey nodes correspond to molecules with similarity below the cutoff. The background color scale (red to blue) indicates the size of the neighboring cluster, measured as the number of molecules it contains.

Among the 47 compounds with predicted p*Ki* values above 9.0, the similarity map (FragFp similarity > 80%) highlights two major clusters together with several structurally diverse candidates, underscoring the presence of multiple chemotypes within the selected set. The largest cluster in the similarity map (green nodes with blue background in Fig 7A) was excluded from further analysis, as the compounds contain ester functionalities that are generally susceptible to hydrolysis under physiological conditions [61]. Among the remaining compounds, the five highlighted as dark blue nodes were selected for further analysis based on their high predicted activity, as detailed in Table 1. In addition, their structural diversity ensured the representation of distinct chemotypes in this group of candidates rather than closely related analogues. Notably, comparison with the 93 reported CrtM inhibitors in the BindingDB database revealed FragFp similarity values below 80% for all five compounds, indicating that they possess distinct structural features not commonly found among known inhibitors (Fig 7B).

**Table 1:** Identities, molecular weight, and predicted inhibitory constants of the selected compounds.

ID	IUPAC name	Mol. Weight	Calculated *pKi*	Calculated *Ki* (nM)
CNP0260941.1	(2R,3R,4S,6S)-3-hydroxy-2,4-dimethyl-5-oxo-6-(3,5,6-trimethyl-4-oxo-pyran-2-yl)heptanoic acid	324.16	9.47	0.339
CNP0460023.0	[3,5-dihydroxy-4-(3-hydroxy-5-sulfooxy-phenoxy)phenyl] hydrogen sulfate	409.96	9.45	0.355
CNP0203663.1	(3S)-3-hydroxy-3-methyl-5-(4,4,7,8-tetramethyltetralin-6-yl)pentanoic acid	318.22	9.44	0.363
CNP0605304.0	2-[3-(3,4-dihydroxyphenyl)prop-2-enoyl]-2,3-dihydroxy-butanedioic acid	312.04	9.42	0.380
CNP0420418.0	(2-hydroxy-5-(5-hydroxy-7-methoxy-4-oxo-3-sulfooxy-chromen-2- yl)phenyl)hydrogen sulfate	475.97	9.36	0.44

The following descriptions annotated in the COCONUT database [24] summarize the chemical features and reported natural sources of the selected compounds. The compound CNP0260941.1 is a polyketide-derived medium-chain hydroxy acid, naturally occurring in *Oldenlandia corymbosa* and *Thalictrum alpinum*. Extracts from *O. corymbosa* have demonstrated broad-spectrum antibacterial activity, and members of the genus *Thalictrum* have also been associated with antimicrobial effects [62]. CNP0460023.0 is a sulfated polyphenolic metabolite isolated from the brown alga *Pleurophycus gardneri*. Although specific antibacterial studies on *P. gardneri* are limited, brown seaweeds are well recognized as prolific sources of antimicrobial metabolites [63]. The compound CNP0203663.1, a botryane-type sesquiterpenoid, features a distinctive tetralin core substituted with a hydroxylated pentanoic acid side chain. This scaffold has been reported in multiple plant including *Artemisia californica* and *Citrus sudachi*. While the genus Artemisia has demonstrated antibacterial activity [64], no studies specifically addressing A. californica were identified. On the other hand, C. sudachi peel extracts have shown inhibitory activity against Vibrio and other foodborne bacteria [65]. CNP0605304.0 belongs to the family of hydroxycinnamic acid derivatives, integrating a 3,4-dihydroxyphenyl moiety with a tartaric acid backbone. This compound has been identified in a broad range of plant species, such as *Vitis longii*, *Callisia fragrans*, and *Echinacea purpurea*—the latter two recognized for their antimicrobial activity [66,67]. Finally, CNP0420418.0 corresponds to Rhamnetin 3,3′-disulfate, a sulfated flavonol isolated from the climbing plant *Argyreia mollis*. Although antibacterial data for this species are scarce, antimicrobial activity has been demonstrated in other members of the genus *Argyreia* [68]. Overall, several of the natural sources associated with our top-ranked compounds have shown antimicrobial properties, yet none have been assessed for CrtM inhibition in *S. aureus*.

It is important to note that some natural products reported to suppress STX synthesis, such as flavones [8] anthraquinones and chalcones [10] were not included in the generation of the pharmacophore and QSAR models, as their activity has not been experimentally confirmed as a direct inhibition of CrtM. Nonetheless, in our analyses, their scaffolds appeared among some of the 47 top-ranked compounds identified in the virtual screenings. In particular, six molecules with a flavone scaffold (CNP0420418.0, CNP0309607.0, CNP0143044.0, CNP0209131.0, CNP0359090.0 and CNP0224661.0); one with an anthraquinone core (CNP0353630.0) and two with chalcone-like hydroxycinnamic backbone (CNP0605304.0 and CNP0572040.0) were identified among these candidates. These findings suggest that CrtM inhibition may underlie, at least in part, the activity of these reported compounds, highlighting the need for further studies to evaluate this potential mechanism of action.

### In silico toxicity and drug-likeness evaluation of the best-scoring CrtM candidates

*In silico* toxicity predictions for the five best-scoring compounds indicated overall favorable safety profiles (S7 Table). None of them showed mutagenic potential in the Ames test, nor did they inhibit the hERG I or II cardiac ion channels, both of which are critical safety endpoints in drug discovery [69,70]. Additionally, no skin sensitization alerts were detected across the dataset.

Among these molecules, four (CNP0420418.0, CNP0460023.0, CNP0203663.1, and CNP0605304.0) were predicted to be non-hepatotoxic, while CNP0260941.1 showed a potential hepatotoxic liability. This compound should therefore be considered with caution in further optimization steps, as structural modifications may be necessary to mitigate this potential hepatotoxic risk.

Regarding systemic toxicity, the five compounds displayed variable tolerance profiles, with predicted maximum tolerated dose (MTD) values ranging from 0.18 to 1.39 log mg/kg/day and chronic oral LOAEL values spanning 1.84 to 4.59 log mg/kg_bw/day. CNP0605304.0 and CNP0460023.0 showed the highest LOAEL values, indicative of lower chronic toxicity and better tolerance under prolonged exposure. In contrast, CNP0260941.1 exhibited the lowest LOAEL and LD₅₀ values, pointing to a higher predicted systemic toxicity within the series.

The evaluation using SwissADME (S8 Table) indicated that the five compounds largely met the main criteria for oral drug-likeness. Most complied with the Lipinski and Ghose rules [71,72], showing suitable molecular size and lipophilicity, while minor deviations in hydrogen bonding or polarity (TPSA > 140 Å²) were observed in CNP0460023.0, CNP0605304.0, and CNP0420418.0. In contrast, CNP0260941.1 and CNP0203663.1 fully satisfied all filters and achieved the highest predicted oral bioavailability scores (0.56 and 0.85, respectively). Synthetic accessibility scores (3.1–4.7) indicated moderate feasibility for chemical synthesis. No relevant PAINS alerts were detected, although CNP0605304.0 contained a catechol moiety and CNP0460023.0/CNP0420418.0 showed sulfonic acid fragments flagged by Brenk filters [73]. Together, these findings highlight CNP0260941.1 and CNP0203663.1 as the most balanced candidates in terms of physicochemical properties and predicted pharmacokinetic behavior.

### Molecular docking, molecular dynamics and energy binding analyses

**Fig 8:**
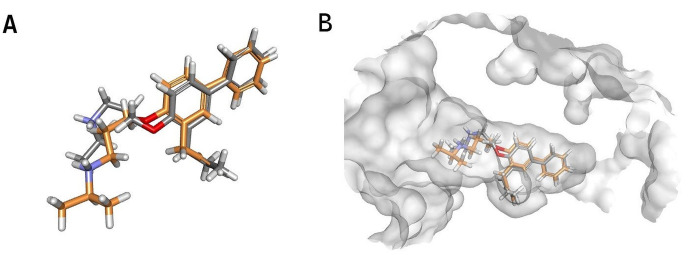
Comparison between the co-crystallized and redocked poses of the 3ACX ligand. **(A)** Superposition of the experimental (tan) and redocked (grey) conformations of the co-crystallized ligand 673, showing close agreement between the two poses. **(B)** Side view of the 3ACX active site displaying both ligand conformations superimposed within the binding pocket.

Molecular docking was performed using the PLANTS software to determine the binding poses of the five top-ranked ligands within the CrtM active site. To validate the docking protocol, the co-crystallized ligand was redocked into the CrtM binding site, yielding a root mean square deviation (RMSD) of 2.33 Å relative to the experimental pose, with only deviations in the flexible side chains (Fig 8). Using the docking poses as starting conformations, the stability of the ligand–enzyme complexes was evaluated through the analysis of RMSD values under dynamic conditions, a widely used criterion for monitoring structural fluctuations over time [74]. Lower and stable RMSD values are generally indicative of strong and persistent binding, while higher or progressively increasing values may signal reduced stability or potential dissociation [75].

**Fig 9:**
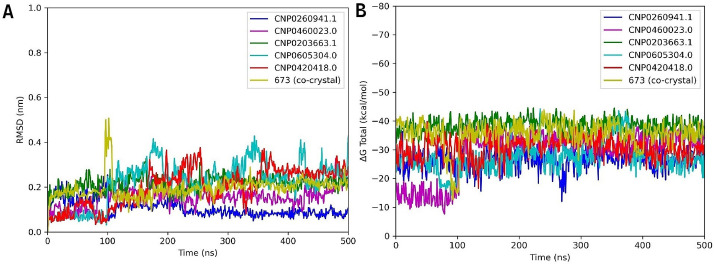
MD analysis of the CrtM complex with the five best-ranked compounds and co-crystallized ligand. **(A)** RMSD values and **(B)** ΔG Total.

The ligands CNP0260941.1, CNP0460023.0, and CNP0203663.1 maintained RMSD values below 0.3 nm for most of the simulation, indicating that they remained stably associated with the binding site. Among them, CNP0260941.1 showed particularly stable behavior: from 215 ns to the end of the trajectory its RMSD stayed near 0.1 nm, making it the most stable ligand–enzyme complex in the set. The co-crystallized ligand stabilized around 0.2 nm at the beginning of the run but displayed a sharp, transient increase to about 0.5 nm near 100 ns. It then regained stability, maintaining an RMSD close to 0.2 nm until the end of the simulation. Ligands CNP0420418.0 and CNP0605304.0 remained stable during the first 100 ns but later exhibited some fluctuations, indicating lower overall stability relative to the other compounds (Fig 9A).

The compound with the most favorable binding energy was CNP0203663.1, which showed an average value of –39 kcal/mol during the analysis time, consistent with its stability observed in the RMSD results. CNP0260941.1 and CNP0605304.0 displayed average values of approximately –28 kcal/mol, with similar energetic profiles throughout the simulation. CNP0420418.0 exhibited a binding energy of approximately –30 kcal/mol during the first 30 ns, although with marked fluctuations that coincided with variations in the RMSD values. Finally, CNP0460023.0 displayed a less favorably binding energy at the beginning of the run, with a mean value of approximately –16 kcal/mol. However, from 100 ns onward, the binding energy improved to around –30 kcal/mol, which may account for the reduction in the RMSD values observed, (Fig 9B).

**Fig 10:**
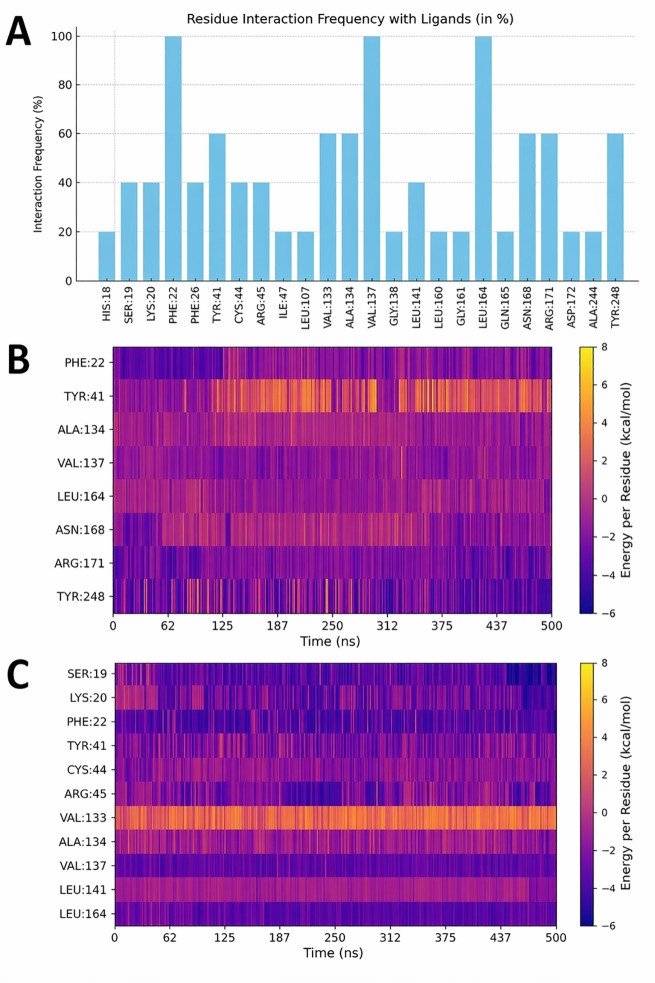
Residue interaction frequency and energy decomposition analysis for CrtM complexes. **(A)** Frequency of interactions between CrtM active-site residues and co-crystallized ligands, as reported in the Protein Data Bank in Europe – Knowledge Base (PDBe-KB). Per-residue energy decomposition of CrtM interactions with compounds **(B)** CNP0260941.1. and **(C)** CNP0460023.0.

The analysis of the energetic contributions of individual interacting residues in the five ligand–CrtM complexes consistently highlighted key interactions of all ligands with PHE22, VAL137, and LEU164. Notably, as show the Fig 10A, these residues are also recurrently involved in interactions with all co-crystallized ligands, as annotated in the PDBe-KB interaction data. In the case of CNP0260941.1, the heatmap shows interactions with eight amino acids (Fig 10B). Favorable interactions were observed with nearly all residues throughout the simulation, with the exception of TYR41, which consistently exhibited unfavorable contacts. In addition to the three amino acids—PHE22, VAL137, and LEU164—common to all the described ligands, ARG171 also contributed significantly to binding. Each of these residues exhibited interaction energies between −2 to −6 kcal/mol. Notably, ARG171 is involved in interactions with other co-crystallized ligands, appearing in 60% of the PDBe-KB complexes (Fig 10A). The CNP0460023.0 ligand interacts with 11 amino acids, each of which is also involved in interactions with at least 40% of the co-crystallized ligands. Most of these residues contributed to binding affinity throughout the majority of the analysis period, with the exception of VAL133 and, to a lesser extent, LEU141, which exhibited deleterious effects on complex stability (Fig 10C).

**Fig 11:**
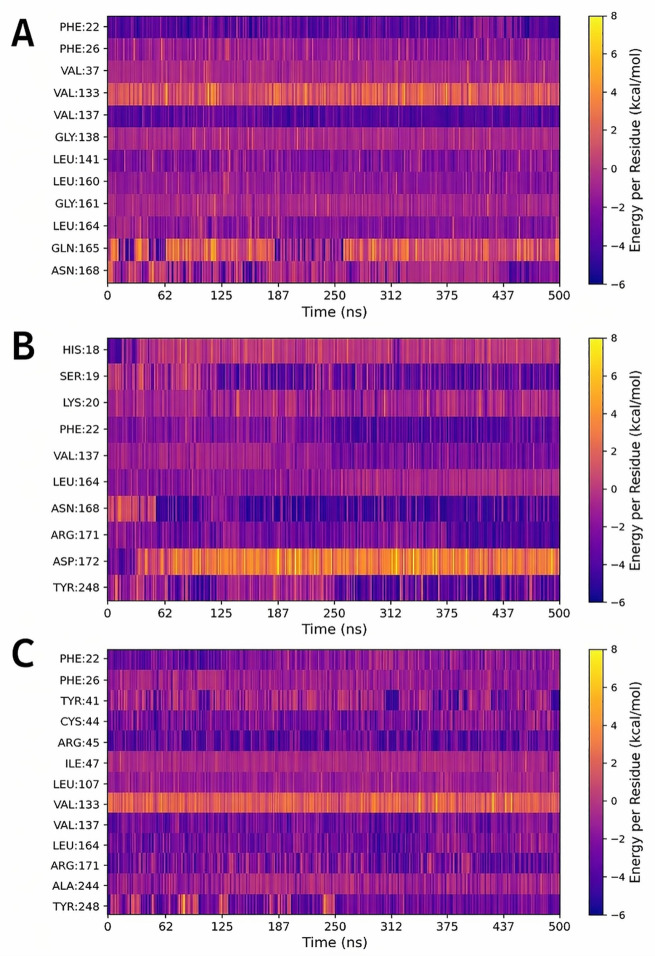
Per-residue energy decomposition of CrtM interactions with compounds (A) CNP0203663.1 (B) CNP0605304.0 and (C) CNP0420418.0.

CNP0203663.1 interacts with 12 residues (Fig 11A); however, unlike in the other cases, several of these residues—such as GLY138, LEU160, GLY161, and GLN165—are less commonly involved in crystallographic complexes, showing a frequency of about 20%. Furthermore, this ligand establishes contacts with VAL37, an amino acid not previously observed in interactions with co-crystallized ligands. Throughout the MD run, most of the ligand–residue contacts played a notable role in stabilizing the ligand–protein complex, which explains its high binding affinity, despite VAL133 and GLN165 exhibiting a detrimental effect on binding affinity. CNP0605304.0 exhibited 10 interactions, with contacts involving SER19, PHE22, ASN168, and TYR248 demonstrating particular significance due to their considerable energetic contributions (approximately –4 kcal/mol) sustained from approximately 50 ns until the end of the simulation). On the other hand, contacts with HIS18 and ASP172 identified in our analyses, have been reported at a relatively low frequency in CrtM complexes (approximately 20%). Notably, ASP172 exerted the most detrimental effect throughout most of the simulation (Fig 11B). Lastly, CNP0420418.0 formed the greatest number of contacts; with interactions contributing approximately –2 kcal/mol during the majority of the simulation. Additionally, VAL133 was identified as a key residue exhibiting unfavorable interactions that negatively affect the binding affinity (Fig 11C). Overall, these results from molecular dynamics simulations and binding free energy calculations, confirm the stability and affinity profile of all five potential CrtM inhibitors identified through pharmacophore- and QSAR-based screenings, further demonstrating the robustness of our protocols in reliably selecting promising drug candidates.

## Conclusion

In this study, we report a set of 1,978 natural-origin compounds with predicted *Ki* values below 100 nM, which constitutes a broad list of compounds with potential inhibitory activity against CrtM. Among them, 47 top-ranked candidates displayed predicted *Ki* values below 1 nM, highlighting their potential as some of most promising inhibitors identified to date. Furthermore, the presence of molecular scaffolds previously associated with suppression of STX synthesis among some of the top-ranked compounds supports the validity of our screening protocols and suggests that they may be capturing biologically relevant features for targeting this enzyme. In fact, the favorable binding energies and stable molecular interactions of the five representative ligands with the CrtM binding site provide further support for this possibility. Importantly, the substantial number and structural diversity of the predicted candidates expand the chemical space of potential inhibitors, providing a valuable resource for experimental validation and for advancing the rational design of effective STX biosynthesis inhibitors.

## Supporting information

S1 Table CrtM inhibitors and their Ki values obtained from BindingDB. (XLSX)

S2 Table Structures, Ki, and pKi values of CrtM inhibitors obtained from BindingDB. (XLSX)

S3 Table Validation parameters for pharmacophoric modeling using Psearch software. (XLSX)

S4 Table List of compounds filtered by pharmacophoric characteristics. (XLSX)

S5 Table QSAR model validation results. (XLSX)

S6 Table Predicted activity of compounds falling within the applicability domain and activity range. (XLSX)

S7 Table In silico toxicity evaluation of the best-scoring CrtM candidates. (XLSX)

S8 Table Drug-likeness, medicinal chemistry, and pharmacokinetic profiles of the top-scoring CrtM inhibitor candidates. (XLSX)
